# Altered gut microbiota and metabolites in untreated myasthenia gravis patients

**DOI:** 10.3389/fneur.2023.1248336

**Published:** 2023-09-15

**Authors:** Xiao-Jun Ding, Hong-Yan Li, Huaiping Wang, Xue-Hua Zhang, Min Song, Xiao-Han Jiang, Xu Zhang, Yao-Xian Yue, Xiao-Hong Li

**Affiliations:** ^1^Department of Neurology, Qilu Hospital (Qingdao) of Shandong University, Qingdao, China; ^2^Department of Geriatric Medicine, Qingdao Fuwai Cardiovascular Hospital, Qingdao, China; ^3^Department of Clinical Psychology, Affiliated Hospital of Qingdao University, Qingdao, China; ^4^Department of Neurology, Jinan Central Hospital, Shandong University, Jinan, China

**Keywords:** gut microbiota, myasthenia gravis, 16S rRNA, *Faecalibacterium*, metabolites

## Abstract

**Objective:**

The homeostasis of the immune system is influenced by the gut microbiota. Previous studies have reported dysbiosis in the gut microbiota of myasthenia gravis (MG) patients. To investigate potential alterations in gut microbiota and metabolites in newly diagnosed and untreated MG patients, we conducted a case-control study.

**Methods:**

Fecal samples were collected from 11 newly diagnosed and untreated MG patients as well as 11 age-and sex-matched healthy controls. These samples underwent analysis for gut microbiota using 16S ribosomal RNA (rRNA) gene sequencing, while fecal metabolome was analyzed using liquid chromatography-electrospray tandem mass spectrometry system (LC-ESI-MS/MS).

**Results:**

The microbial community richness (observed species) and diversity (Shannon and Simpson indices) were significantly lower in the MG group compared to the control group. Microbiota composition analysis revealed significant differences between the MG and control groups at phylum, family, and genus levels. Linear discriminant analysis effect size (LEfSe) analysis showed a substantial decrease in abundance of the genus *Faecalibacterium* within the MG group. Fecal metabolome analysis identified three up-regulated metabolites involved in amino acid metabolism (taurine, creatinine, L-carnitine), one up-regulated metabolite involved in lipid metabolism (oleic acid), with correlation analysis indicating a positive association between *Faecalibacterium* abundance and creatinine levels.

**Conclusion:**

Our findings suggest that dysbiosis already exists in newly diagnosed and untreated MG patients, implying that dysbiosis within the gut microbiota may be an initiating factor contributing to MG pathogenesis. Furthermore, *F. prausnitzii* may hold promise as a probiotic for treating MG.

## 1. Introduction

Myasthenia gravis (MG) is an antibody-mediated autoimmune disease characterized by fluctuating muscle weakness and fatigue. Pathogenic antibodies targeting the acetylcholine receptor (AChR), muscle-specific tyrosine kinase (MuSK), and lipoprotein receptor-related protein 4 (LRP4), impair the conduction of the neuromuscular junctions (NMJ) of the postsynaptic membrane (1). Previous studies (2–6) had reported the dysbiosis in the gut microbiota between MG patients and healthy controls (CO), their metabolites notably differ in metabolic pathways such as amino acid, lipid, and nucleotide. Germ-free (GF) experimental autoimmune myasthenia gravis (EAMG) mice colonized with MG patients’ microbiota had substantially impaired locomotion ability compared to mice colonized with COs’ microbiota, and this effect could be reversed by co-colonizing GF mice with both MG and COs’ microbiota (3). Gut microbiota and their metabolites damage the intestinal mucosa, increase permeability, and disrupt the balance of Th17/Treg cells contributing to the pathogenesis of MG (7).

Several studies have reported a correlation between gut microbiota and MG; however, these results may be affected by confounding factors such as immunosuppressant drugs or co-morbid diseases. The novelty of this study is that we recruited newly diagnosed untreated MG patients who were native to immunosuppressive therapy, both MG and CO group had no previous disease histories or medication within 3 months prior to enrollment. We used 16S ribosomal RNA (rRNA) gene sequencing and metabolomics studies to explore alterations in gut microbiota and metabolites in MG patients while discussing probiotics as potential therapeutic approaches for treating MG.

## 2. Materials and methods

### 2.1. Human subjects and sample/data collection

Newly diagnosed MG patients who received treatment and follow-up at Qilu Hospital (Qingdao) of Shandong University between November 2018 and October 2020 were included in this study. General clinical features, including gender, age of onset, presence of thymoma (confirmed by chest CT and/or pathology), AChR and MuSK antibodies, Myasthenia Gravis Foundation of America (MGFA) classification at onset and sampling, as well as treatment drugs were recorded. Healthy individuals undergoing check-ups at our hospital were recruited as controls. Both the MG patients and controls denied any previous history of diseases such as gastrointestinal tract disorders, coronary heart disease, stroke, hypertension, diabetes mellitus, malignancy tumors or other autoimmune diseases. None of the participants had taken antibiotics, glucocorticoids or probiotics within 3 months prior to this study. The study protocol was approved by the ethical committee of Qilu Hospital (Qingdao) of Shandong University. Informed consent was obtained from both MG patients and controls.

### 2.2. Fecal specimen collection, DNA extraction, and sequencing

We provided each participant with a sterilized container for stool collection. All participants underwent formal training on sample collection at the time of recruitment. Fecal samples were collected immediately, transported to the laboratory, and promptly frozen at −80°C. Total genomic DNA was extracted using the OMEGA Soil DNA Kit (M5635-02) (Omega Bio-Tek, Norcross, GA, United States). PCR amplification of the V3-V4 region of bacterial 16S rRNA genes was performed using forward primer 338F (5′-ACTCCTACGGGAGGCAGCA-3′) and reverse primer 806R (5′-GGACTACHVGGGTWTCTAAT-3′). PCR amplicons were purified with Vazyme VAHTSTM DNA Clean Beads (Vazyme, Nanjing, China) and quantified using the Quant-iT PicoGreen dsDNA Assay Kit (Invitrogen, Carlsbad, CA, United States). After individual quantification steps, amplicons were pooled in equal amounts and subjected to pair-end 2*250 bp sequencing on the Illumina NovaSeq platform with NovaSeq 6000 SP Reagent Kit at Shanghai Personal Biotechnology Co., Ltd. (Shanghai, China).

### 2.3. Bioinformatics and statistical analysis

QIIME2 and the R package (v3.2.0) were primarily utilized for our sequence data analysis. The α-diversity indices at the amplicon sequence variant (ASV) level, including observed species, Shannon diversity index, and Simpson index, were computed using the ASV table in QIIME2 and visualized through box plots. To examine the structural variations of microbial communities across different samples, β-diversity analysis was conducted employing Bray-Curtis metrics and visualized via Principal Coordinate Analysis (PCoA). A Venn diagram illustrating shared and unique ASVs between groups was generated based on ASV occurrence in the samples, irrespective of their relative abundance, utilizing the R package “VennDiagram” (8). Linear discriminant analysis effect size (LEfSe) with default parameters was employed to identify taxa with differential abundance between groups (9). Statistical analyses were performed using SPSS 23.0 software. Continuous variables such as age were compared using Student’s *t*-test while categorical variables like gender were analyzed using Chi-square test. *p* < 0.05 denoted statistical significance.

### 2.4. Metabolic profiling and association analysis

The fecal metabolites were subjected to analysis using the HILIC UHPLC-Q-TOF/MS metabolomics analysis method. Subsequently, the raw MS data were converted to MzXML files using ProteoWizard MSConvert and imported into the free XCMS software for further processing. Normalization was performed based on total peak intensity, and the processed data were then uploaded onto SIMCA-P (version 14.1, Umetrics, Umea, Sweden) for multivariate data analysis employing orthogonal partial least squares discriminant analysis (OPLS-DA). Variable importance plot (VIP) values were obtained from OPLS-DA model analysis. Differentially expressed metabolites (DEMs) were selected based on VIP > 1 and *p*-values (<0.05) derived from Student’s *t*-test conducted on normalized peak areas. The identified metabolites underwent metabolic pathway analysis utilizing the Kyoto Encyclopedia of Genes and Genomes (KEGG) database.^1^ Correlation analyses were performed using Mental Test and Spearman correlation methods. Significance was determined at *p*-values below 0.05: *p ≤ 0.05 and **p ≤ 0.01.

## 3. Results

### 3.1. General characteristics

**Figure 1:**
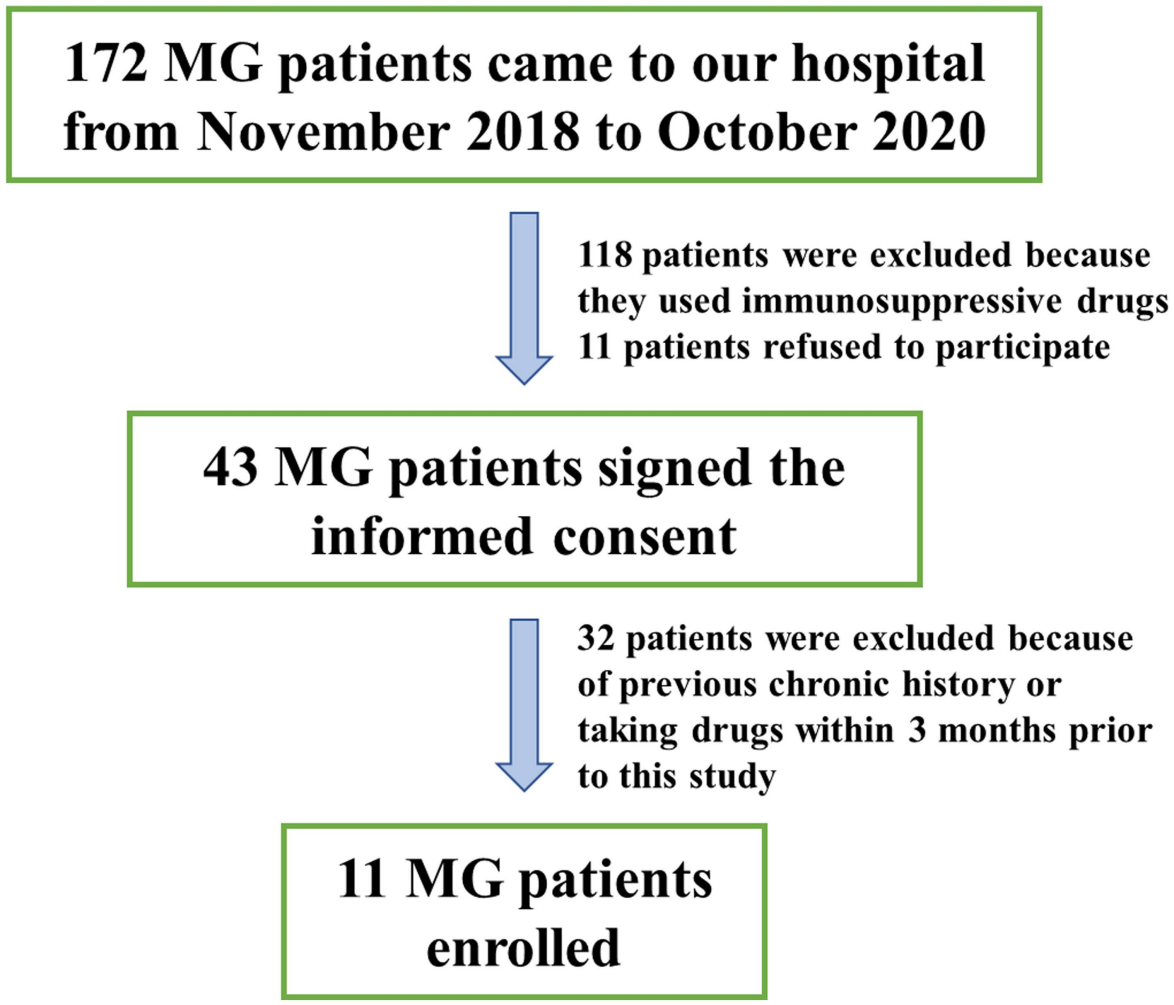
Enrollment profile.

A total of 11 MG patients and 11 COs were recruited in the study, as depicted in Figure 1. The MG group consisted of 4 males and 7 females, with a mean age at sampling of 51.82 ± 9.24 years and a body mass index (BMI) of 27.15 ± 3.54. The CO group included 6 males and 5 females, with a mean age at sampling of 52.91 ± 11.74 years and a BMI of 25.94 ± 5.20. There were no statistically significant differences observed between the two groups in terms of age or BMI at the time of sampling (Table 1).

**Table 1:** Characteristics of MG patients.

Patient	Gender	BMI	Onset age	Thymoma	Antibody	MGFA classification at onset	MGFA classification at sampling
1	Female	27.69	38	None	DSN	IVa	IVa
2	Male	30.49	63	None	AChR	I	I
3	Female	28.58	54	Thymolipoma	AChR	I	I
4	Female	31.99	51	None	AChR	IIb	IIb
5	Female	22.04	68	None	DSN	I	I
6	Female	24.98	51	None	MuSK	IIb	IIb
7	Male	31.92	49	None	AChR	I	IIa
8	Female	26.35	48	None	AChR	I	I
9	Male	26.13	47	None	AChR	I	IIa
10	Male	27.12	40	None	AChR	I	IIb
11	Female	21.37	61	None	AChR	IIIa	IIIa

BMI, body mass index; DSN, double (both AChR and MuSK) seronegative.

### 3.2. Overall structure of bacterial communities across samples

**Figure 2:**
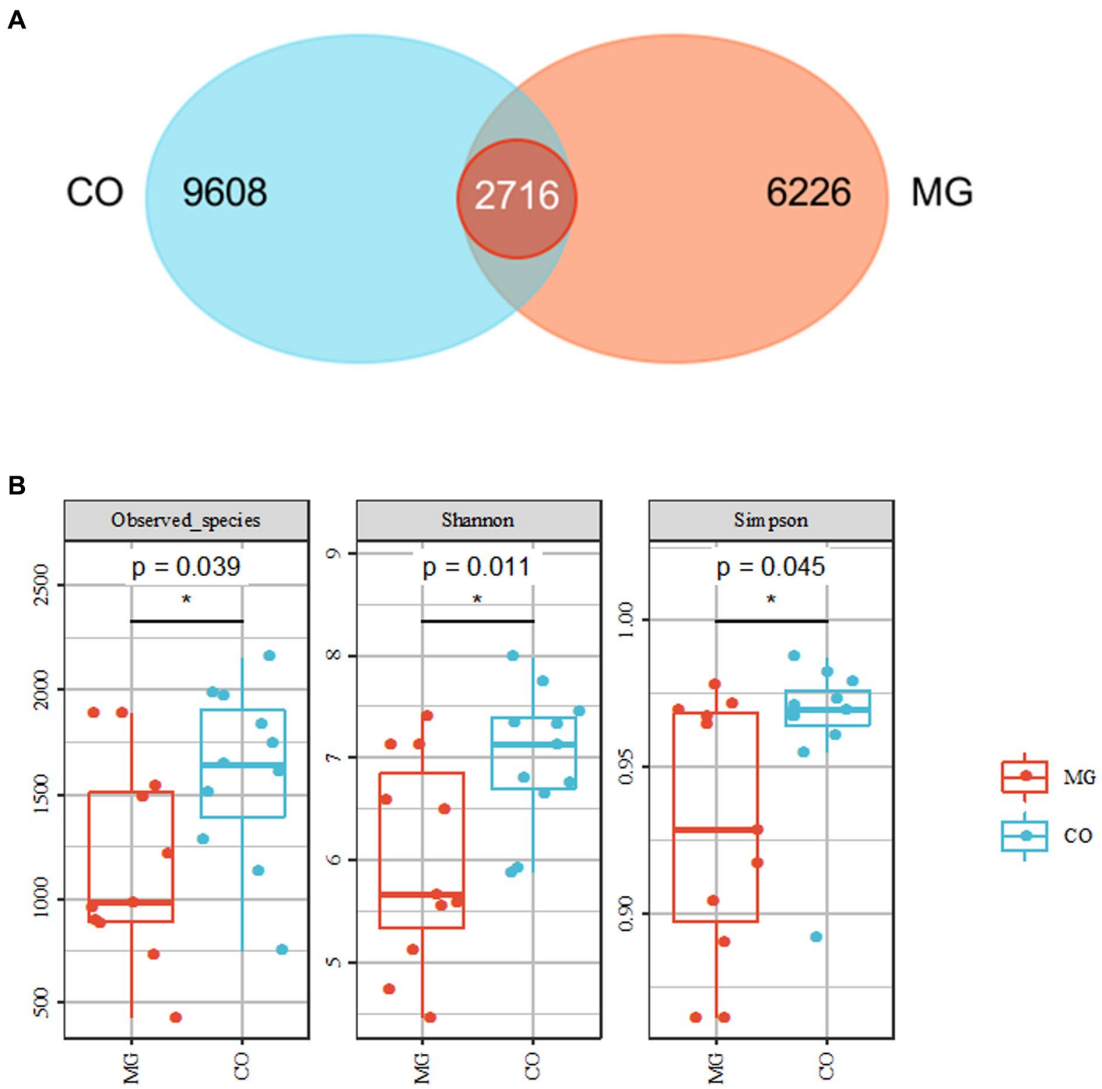
Overall structure of bacterial communities across samples. **(A)** Venn diagram shows that 2,716 amplicon sequence variants (ASVs) were co-present in the MG and CO groups and 9,608 were present only in the MG group, while 6,626 were unique to CO group. **(B)** α-diversity analysis revealed that microbial richness and diversity were significantly reduced in MG group compared to CO group.

We identified a total of 1,768,258 high-quality sequences in 22 samples through 16S rRNA gene sequencing. These sequences were clustered into 19,447 ASVs with a sequence similarity of 100%. The ASVs were quantified separately at the phylum to genus level. To analyze the community composition, we generated a Venn diagram using the ASV richness table and found that there were 2,716 shared ASVs between the MG and CO groups. Additionally, there were 9,608 and 6,266 unique ASVs in the MG and CO group, respectively (Figure 2A). In order to compare the α-diversity of bacterial communities within samples between these two groups based on ASV levels, multiple indices including observed species richness as well as Shannon and Simpson diversity indices were utilized (10). Our results demonstrated that MG patients had significantly lower values for observed species richness as well as Shannon and Simpson diversity indices compared to COs (Figure 2B).

### 3.3. Microbiota composition analysis of MG and CO groups

**Figure 3:**
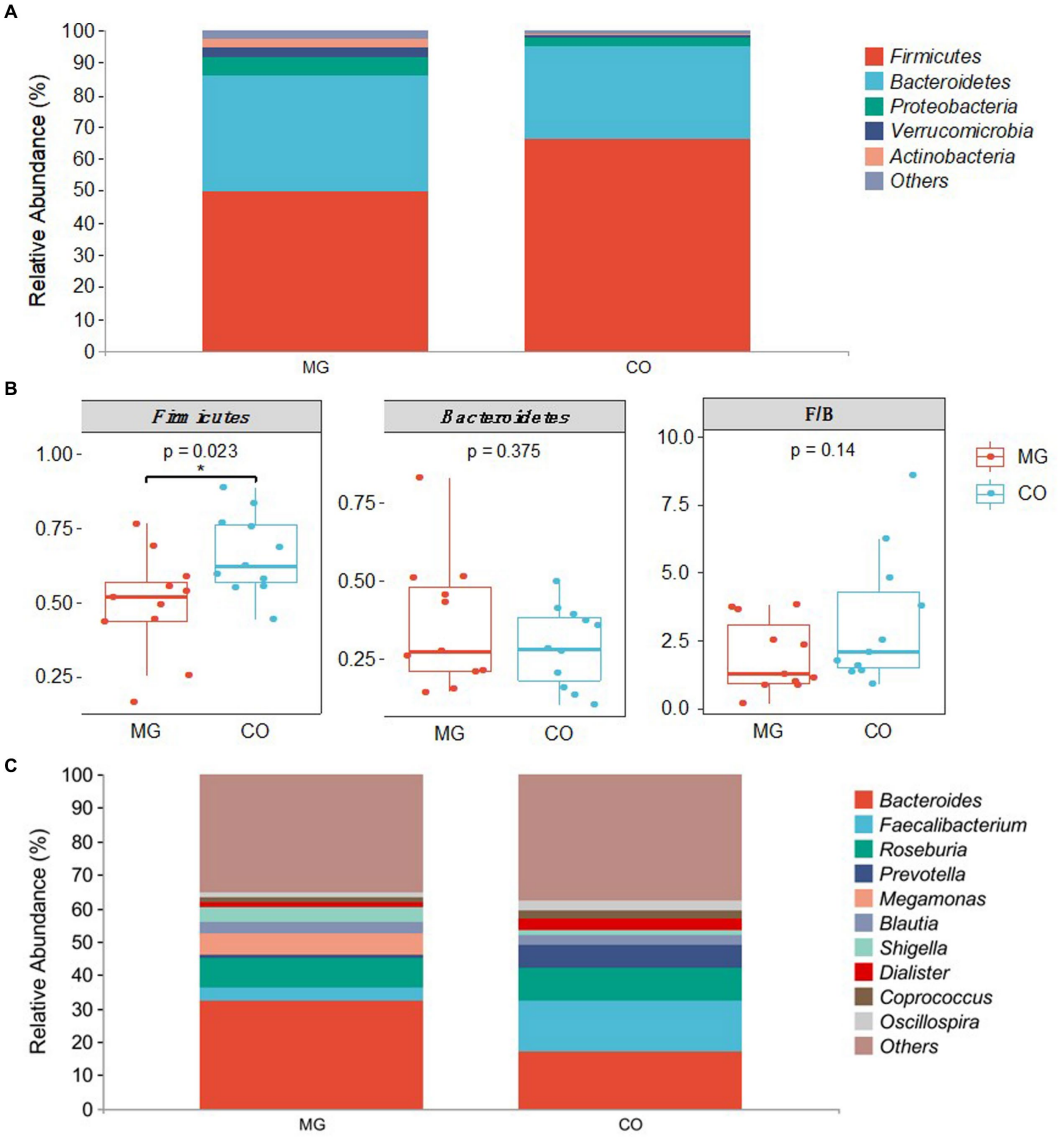
Differences in gut microbiota composition between MG and CO group. **(A)** Microbiota composition of samples from MG and CO group at the phylum level. **(B)** Mean ± SEM of relative abundance of different phyla. *p < 0.05 by unpaired *t*-test. Abbreviation: *F*: *Firmicutes, B: Bacteroidetes.* **(C)** genus-level composition of individuals from MG and CO groups.

The gut microbiota composition at the phylum and genus levels for the MG and CO groups is presented in Figure 3. At the phylum level, *Firmicutes* and *Bacteroidetes* were found to be the dominant phyla in both MG and CO groups’ gut microbiota composition, followed by *Proteobacteria*, *Verrucomicrobia,* and *Actinobacteria* (Figure 3A). The abundance of *Firmicutes* was significantly reduced in the MG group compared to the control group, resulting in a decreased *Firmicutes/Bacteroidetes* ratio (Figure 3B). A comparison of genus-level components between these two groups was shown in Figure 3C. In the MG group, there was an increase in abundance of *Bacteroides, Megamonas, Blautia*, and *Shigella* at the genus level compared to the control group. Conversely, there was a decrease in abundance of *Faecalibacterium, Roseburia Prevotella Dialisster Coprococcus Oscillospira* genera.

**Figure 4:**
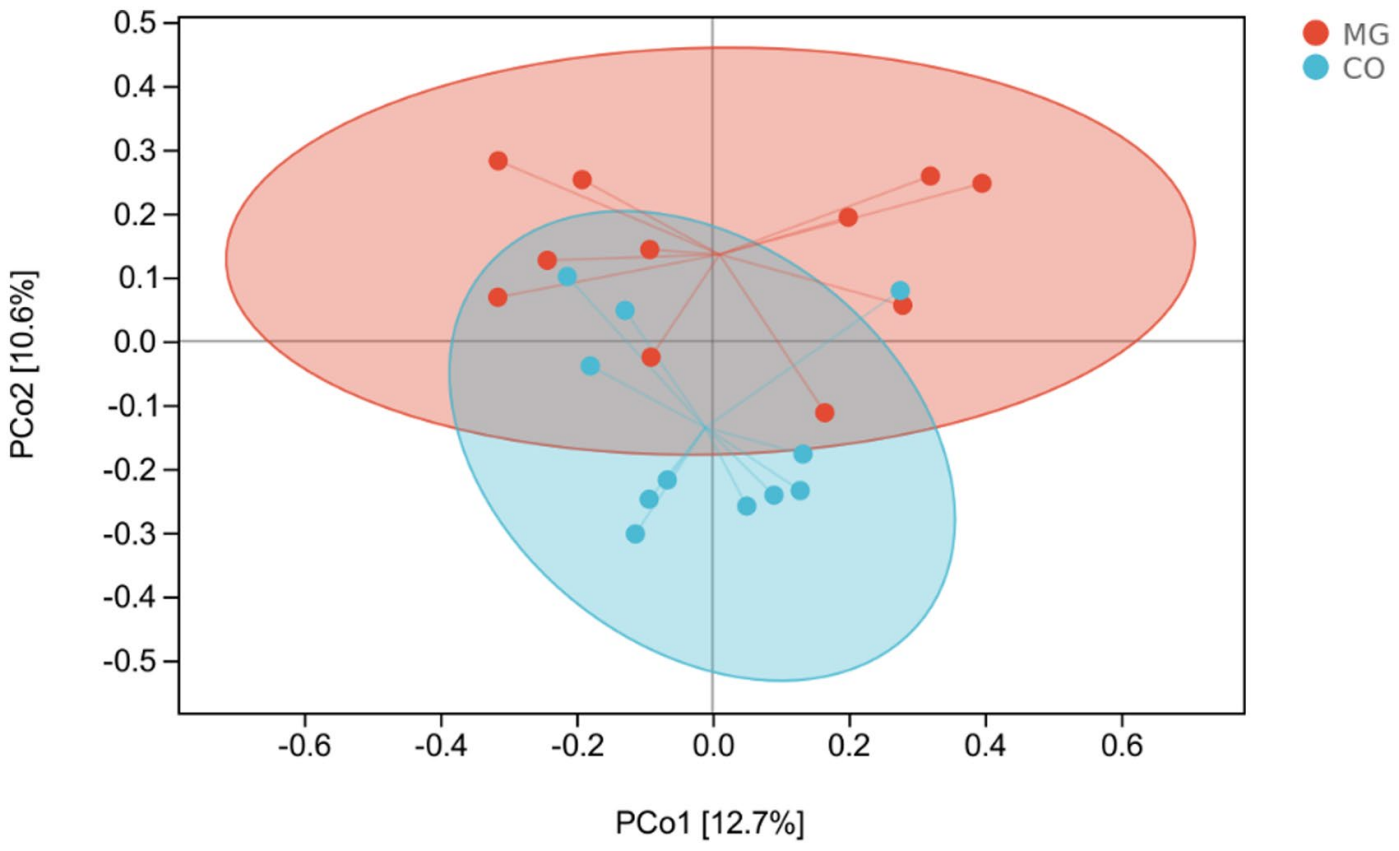
Patterns of the gut microbiota in the MG and control groups differentiated by unsupervised principal coordinate analysis (PCoA), PERMANOVA, *p* = 0.003.

β-Diversity analysis using PcoA based on Bray-Curtis dissimilarity revealed significant differences in gut microbiota composition between the MG and CO groups (PERMANOVA test: *p* = 0.003; Figure 4).

**Figure 5:**
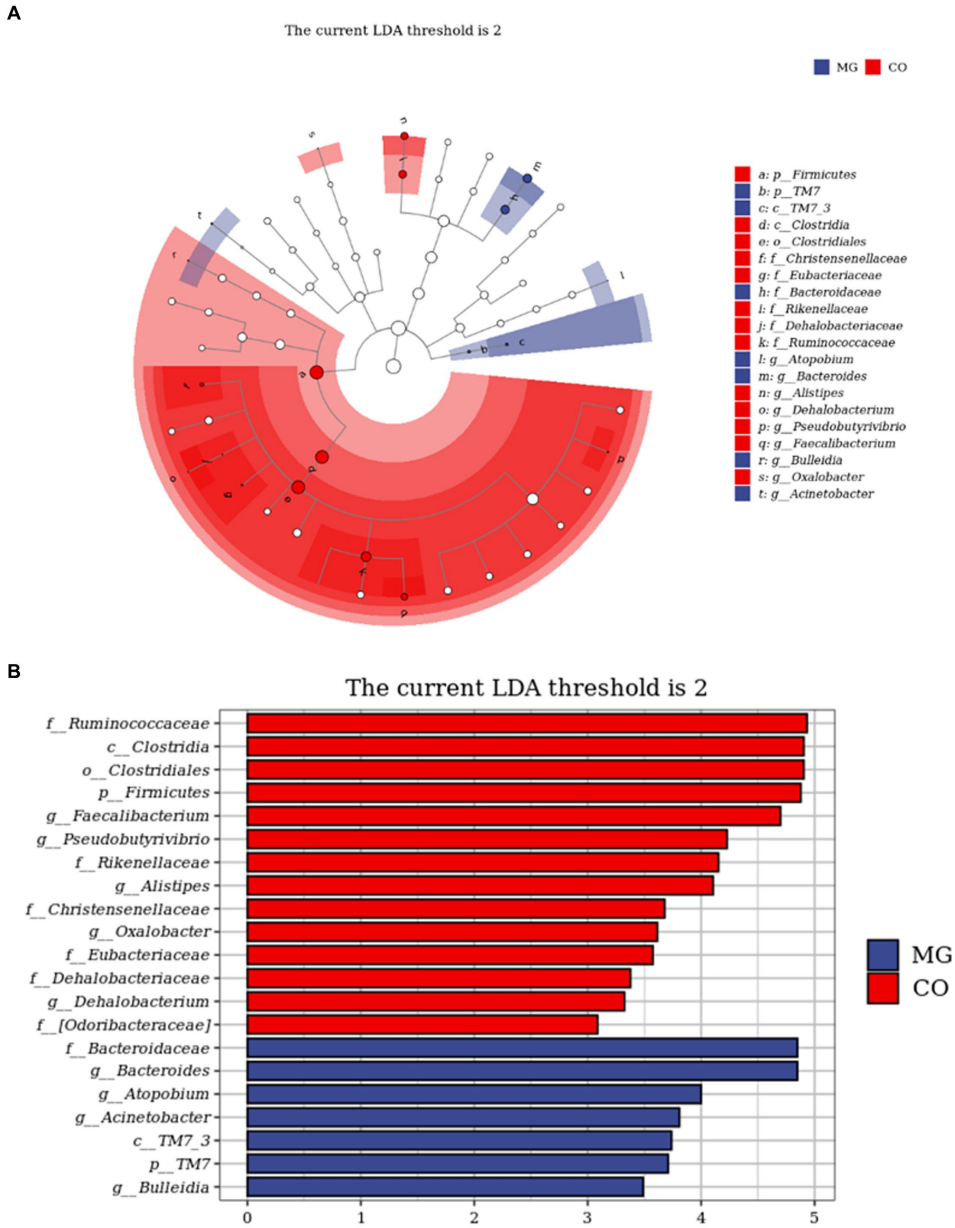
Identification of differential taxa between the two groups by linear discriminant analysis effect size (LEfSe) analysis. **(A)** Cladogram demonstrated the taxonomic hierarchy distribution of the marker species in each group of samples The size of the dots reflects its effect size. **(B)** Taxa that meet the LDA score significance threshold >2.

The LEfSe analysis served as an analytical tool that integrates the nonparametric Kruskal-Wallis and Wilcoxon rank sum tests with linear discriminant analysis (LDA) Effect size. Figure 5A illustrates the species taxonomic branching map (Cladogram), which demonstrates the distribution of marker species in each group based on their taxonomic hierarchy. By employing LDA, we identified 21 distinguishing features (LDA score > 2) that exhibited significant changes in relative abundance between the two groups of fecal samples, as depicted in Figure 5B. According to this histogram, there was a decrease in the abundance of phylum *Firmicutes* (class *Clostria*, order *Clostridiales*, families *Ruminococcaceae, Christensenellaceae, Eubacteriaceae, Dehalobacteriaceae* and genera *Faecallibacterium, Pseudobutyrivibrio, Alistipes, Dehalobacterium*), while an increase was observed in phylum *TM7* (class *TM7_3*) within the MG group. At family and genus levels, we noted an increase in family *Bacteroidetes* (genus *Bacteroides*), genus *Atopobium*, genus *Acinetobacter* and genus *Bulleidia*; conversely a decrease was observed in family *Rikenellaceae* and *[odoribacteraceae]*, as well as genus *Oxalobacter* within the MG group.

### 3.4. Fecal metabolome analysis

**Figure 6:**
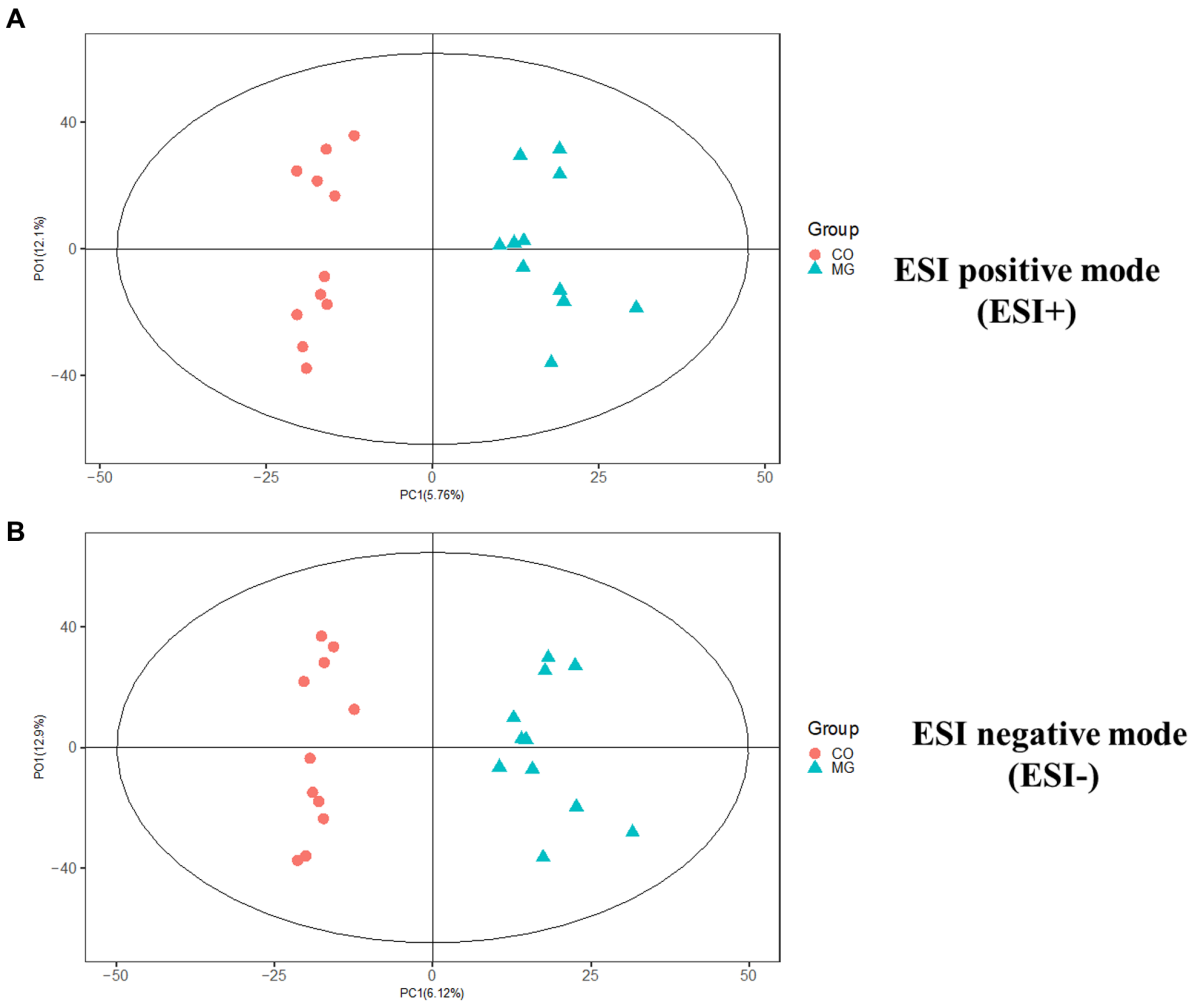
The orthogonal partial least squares discriminant analysis (OPLS-DA) score plot showed a significant difference between MG and CO group in ESI+ and ESI− modes. **(A)** ESI positive mode (ESI+). **(B)** ESI negative mode (ESI−).

**Figure 7:**
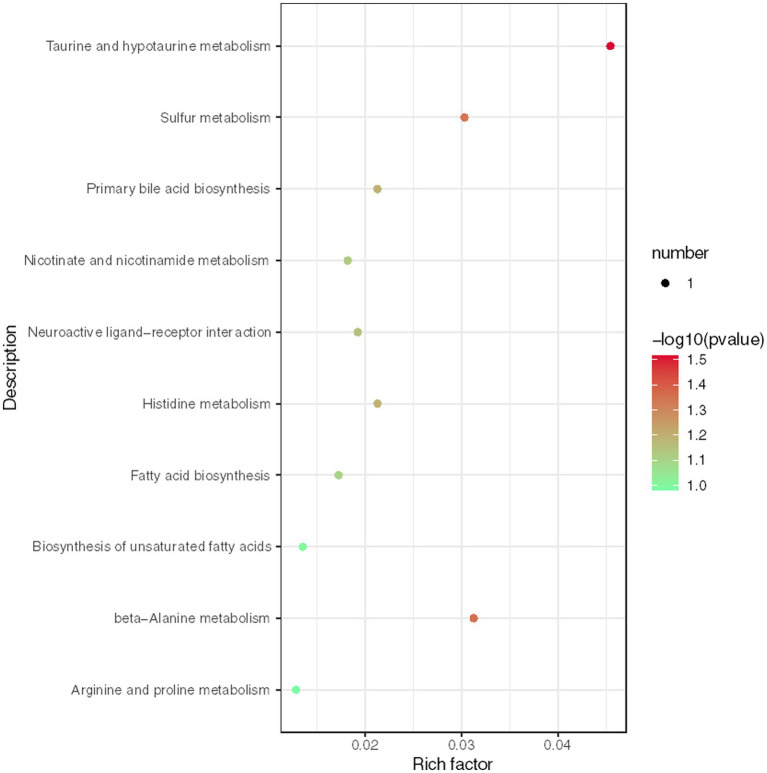
Enrichment analysis of KEGG metabolic pathway reveals key metabolic pathways for differentially expressed metabolites (DEMs).

We conducted further analysis of fecal metabolites using a liquid chromatography-electrospray tandem mass spectrometry system (LC-ESI-MS/MS). OPLS-DA was employed to analyze the clustering information of the MG and CO groups in both ESI positive mode (ESI+) and ESI negative mode (ESI-). The OPLS-DA score plot demonstrated a significant distinction between the MG and CO groups (Figure 6). A total of 19 DEMs were identified in both ESI+ and ESI− modes, with VIP scores greater than 1 and *p* values less than 0.05. Specifically, taurine, pregnenolone sulfate, creatinine, L-phenylalanyl-L-proline, biliverdin, L-carnosine, pyridostigmine cation, oleic acid, and NG, NG-dimethyl-L-arginine (ADMA) were upregulated in the MG group; while tridecanoic acid (tridecylic acid), sebacic acid, alpha-hydroxy myristic acid, Rosolic Acid, 1-Methylpseudouridine, L-Carnosine, N1-Methyl-2-pyridone-5-carboxamide, Harmane, Glycitein, Val-Ala, and Solasodine were down-regulated. Additionally, KEGG enrichment pathway analysis revealed 10 major metabolic pathways from the KEGG database including four amino acid metabolic pathways and three lipid metabolic pathways (Figure 7). Among these pathways, involvement in amino acid metabolism was observed for three up-regulated metabolites (taurine, creatinine, and L-carnitine), while one up-regulated metabolite (oleicacid) was associated with lipid metabolism.

### 3.5. Correlation analysis between DEMs and gut microbiota

**Figure 8:**
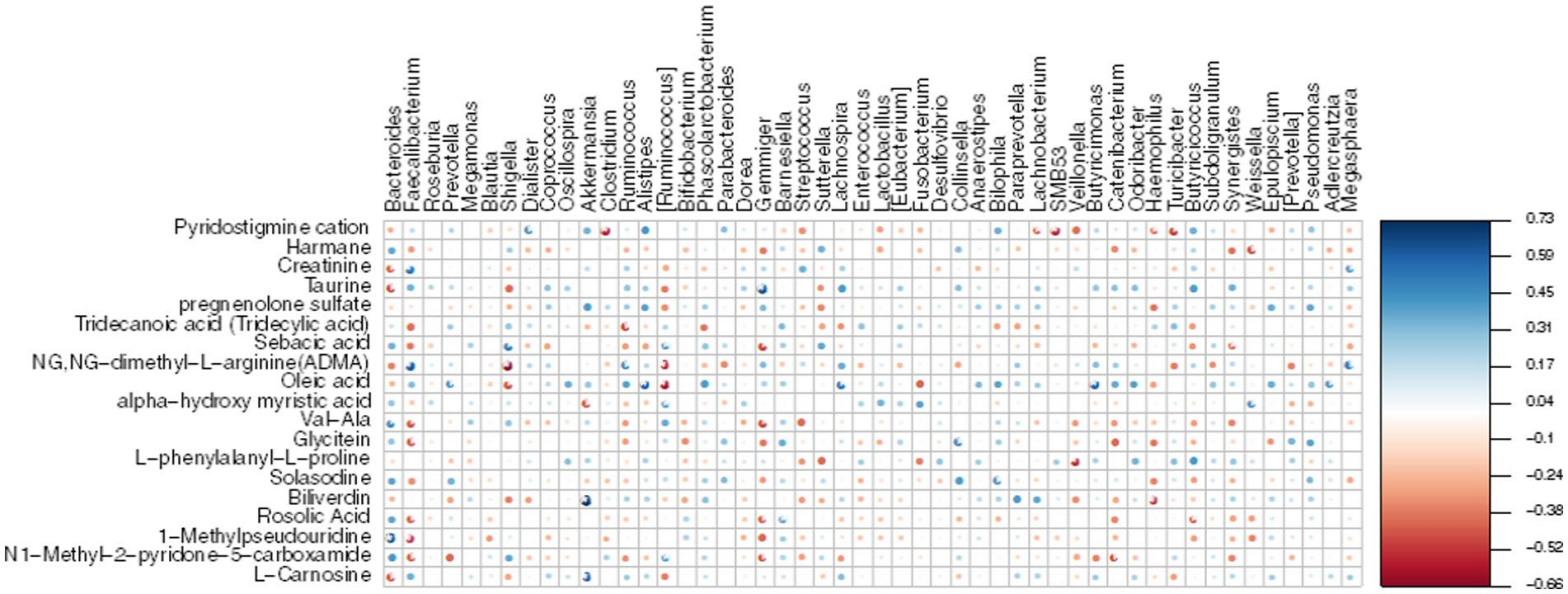
Heatmap displays the Spearman correlation between DEMs and gut microbiota. The *r* values are represented by gradient colors, where red and blue cells indicate negative and positive correlations, respectively; *p < 0.05, **p < 0.001.

To establish the correlation between DEMs and gut microbiota, we conducted a Mental test and Spearman correlation analysis on the relative abundance of specific microbial species and the concentrations of all 19 DEMs in MG and CO groups, primarily focusing on amino acid and lipid metabolism. These metabolite biomarkers exhibited significant associations with gut microbiota (Mental test, *p* = 0.006). *Bacteroides* demonstrated a negative correlation with creatinine, taurine, and L-Carnosine, while *Faecalibacterium* displayed a positive correlation with creatinine (Figure 8).

## 4. Discussion

The intestinal mucosa serves as the largest immune organ within the human body. Alteration of the gut microbiome can result in damage to the intestinal mucosa, leading to increased permeability and an imbalance between Th17 and Treg cells. These alterations have been observed in various autoimmune diseases, including rheumatoid arthritis (11), inflammatory bowel disease (IBD) (12), multiple sclerosis (13), SLE (14), and MG (7).

In this study, we conducted a comparative analysis of the gut microbiota and metabolites in 11 newly diagnosed and untreated MG patients for the first time. Both the MG and control groups had no prior history of diseases or medication within 3 months before enrollment, aiming to minimize the confounding factors such as immunosuppressant drugs and co-morbid conditions. A higher α-diversity is generally associated with better health status (15). Our findings revealed a significant decrease in both α-diversity (diversity within the gut microbiota) and β-diversity (differences between individual microbiotas) in the MG group compared to the control group, indicating existing dysbiosis in newly diagnosed and untreated MG patients. Additionally, we observed a notable decrease in *Firmicutes* proportion accompanied by an increased proportion of *Bacteroidetes* in the gut microbiota of the MG group, consistent with previous studies in MG patients (2–4). Furthermore, we identified a lower *Firmicutes/Bacteroidetes (F/B)* ratio in the MG group compared to the control group. This lower *F/B* ratio suggests a pro-inflammatory environment that can promote immune responses leading to immune imbalance. Similar trends toward lower *F/B* ratios have been reported in autoimmune diseases such as SLE (16) and IBD (17).

Analysis of microbiota composition revealed significant differences at the phylum, family, and genus levels between the MG and CO groups. At the family level, *Bacteroidaceae* was significantly increased in the MG group compared to the CO group, while *Ruminococcaceae* was significantly decreased. Previous studies have shown that *Ruminococcaceae* is a common producer of short-chain fatty acids (SCFAs) (18) and its decrease has been associated with gastrointestinal damage in various diseases such as IBD (19) and lupus nephritis (20). At the genus level, we observed a dramatic decrease in *Faecalibacterium* abundance in the MG group, which is consistent with previous findings (4). The abundance of genera *Pseudobutyrivibrio, Alistipes, Dehalobacterium, and Oxalobacter* was also found to be reduced in the MG group; however, their association with autoimmune diseases has not been reported. *Faecalibacterium*, a crucial component of phylum *Firmicutes*, class *Clostridium*, family *Ruminococcaceae*, was found to be one of the most prevalent species in the intestinal microbiome of healthy adults, constituting approximately 5% of the total bacterial population (21). The present study revealed a significant decrease both in the abundance of family *Ruminococcaceae* and genus *Faecalibacterium* in the MG group. These findings suggest that dysbiosis already exists in newly diagnosed and untreated MG patients, indicating that dysbiosis within the gut microbiota may be an initiating factor contributing to MG pathogenesis.

Until now, *F. prausnitzii* has been identified as the only species within the *Faecalibacterium* genus. It plays a regulatory role in colon cell metabolism and protects intestinal mucosal integrity to maintain intestinal homeostasis and health (22). The decrease in *F. prausnitzii* had been reported in IBD and MS patients (23). *F. prausnitzii* exhibits anti-inflammatory activity by producing various anti-inflammatory metabolites. Additionally, it is one of the most abundant butyrate-producing bacteria in the gastrointestinal tract (GIT) (24), which plays a crucial role in maintaining Th17/Treg homeostasis. Previous studies have demonstrated that butyrate produced by *F. prausnitzii* reduces IL-17 and Th17 production by inhibiting the downstream pathway of IL-6/STAT3/IL-17 through HDAC1 inhibition and activates Foxp3 to enhance Treg cell differentiation (25), thereby maintaining Th17/Treg homeostasis and exerting a pronounced anti-inflammatory effect. In this study, we also observed a significant decrease in *F. prausnitzii* abundance in MG group samples; therefore, it is possible that both *F. prausnitzii* itself and its SCFAs disrupt Th17/Treg cell balance contributing to MG pathogenesis. Further validation studies will be conducted using EAMG models to explore whether *F. prausnitzii* could potentially become a promising probiotic for MG treatment.

Fecal metabolome analysis was conducted to elucidate “what had happened” in the gut of MG and CO groups. In MG group, three up-regulated metabolites (taurine, creatinine and L-carnitine) were involved in amino acid metabolism; one up-regulated metabolite (oleic acid) was involved in lipid metabolism. Consistent with our results, Tan et al. (5) also reported altered fecal amino acid metabolism in MG patients. However, the exact correlations between these metabolites (taurine, creatinine, L-carnitine, and oleic acid) in either blood or feces have not been previously reported in relation to MG. Additionally, we investigated the correlation between 19 DEMs and gut microbiota and observed a negative correlation between *Bacteroides* and Creatinine, Taurine, as well as L-Carnosine. Conversely, *Faecalibacterium* exhibited a positive correlation with Creatinine. Based on these findings, we propose a hypothesis that alterations in gut microbiota including decreased levels of *F. prausnitzii* may increase susceptibility to MG and affect metabolite levels through amino acid metabolic pathways.

There are some limitations in this study. Firstly, the small sample size of MG and control group limited the ability to describe the subgroup correlations with gut microbiota and metabolites. Secondly, we did not detect the gut microbiota and metabolites after immunosuppressive therapy, lacking the potential association between alterations in gut microbiota and metabolites and treatment response.

## 5. Conclusion

Our findings suggest that dysbiosis already exists in newly diagnosed and untreated MG patients, implying that dysbiosis within the gut microbiota may be an initiating factor contributing to MG pathogenesis. Furthermore, *F. prausnitzii* may hold promise as a probiotic for treating MG.

## Data availability statement

The data presented in the study are deposited in the BioProject repository, accession numbers PRJNA990840.

## Ethics statement

The studies involving humans were approved by ethical committee of Qilu hospital (Qingdao) of Shandong University. The studies were conducted in accordance with the local legislation and institutional requirements. The participants provided their written informed consent to participate in this study.

## Author contributions

Y-XY and X-HL conceptualized and designed the study, and revised the manuscript. X-JD interpreted the data and wrote the manuscript. H-YL, XZ, and HW performed statistical analysis. X-HZ, X-HJ, and MS maintained the research database. All authors contributed to the article and approved the submitted version.

## Funding

This study was supported by Qingdao Key Health Discipline Development Fund, Qingdao Clinical Research Center for Rare Neurological Disorder (22-3-7-lczx-3-nsh), Research Grant from Qilu Hospital (Qingdao) of Shandong University (QDKY2018ZD01 and QDKY2021RX06 to Y-XY).

## Conflict of interest

The authors declare that the research was conducted in the absence of any commercial or financial relationships that could be construed as a potential conflict of interest.

## Publisher’s note

All claims expressed in this article are solely those of the authors and do not necessarily represent those of their affiliated organizations, or those of the publisher, the editors and the reviewers. Any product that may be evaluated in this article, or claim that may be made by its manufacturer, is not guaranteed or endorsed by the publisher.

## Footnotes

^1^ http://www.genome.jp/kegg/
